# Primary Mediastinal Synovial Sarcoma Presenting as Superior Vena Cava Syndrome: A Rare Case Report and Review of the Literature

**DOI:** 10.1155/2015/651813

**Published:** 2015-05-25

**Authors:** Irappa Madabhavi, Pritam Kataria, Apurva Patel, Swaroop Revannasiddaiah, Asha Anand, Harsha Panchal, Sonia Parikh, Malay Sarkar, Gaurang Modi, Rahul Kulkarni, Sandip Shah

**Affiliations:** ^1^GCRI, Civil Hospital Complex, Asarwa, Ahmedabad, Gujarat 830016, India; ^2^Department of Radiotherapy & Clinical Oncology, Swami Rama Cancer Hospital & Research Institute, Haldwani, Uttarakhand, India; ^3^Department of Pulmonary Medicine, IGMC, Shimla, Himachal Pradesh, India

## Abstract

Primary mediastinal sarcomas are aggressive tumors with a very rare incidence. This report describes the case of a 35-year-old male patient who presented with acute symptoms of dyspnoea, facial puffiness, voice-hoarseness, and engorged neck veins. With the clinical picture consistent with the superior vena cava (SVC) syndrome, the patient was investigated with computed tomography of the chest. This revealed a large soft tissue density mass lesion compressing the SVC along with other critical superior mediastinal structures. Histopathological evaluation of the mass revealed features consistent with a soft tissue sarcoma and positive staining was observed for vimentin and S-100. Cytogenetic analysis by fluorescent in situ hybridisation (FISH) demonstrated the t(X:18) translocation. Thus diagnosis was established as primary mediastinal synovial sarcoma. Patient was treated with three cycles of neoadjuvant chemotherapy, to which there was a partial response as per the *RECIST* criteria. Surgical excision of the mediastinal mass was performed, and further postoperative treatment with adjuvant chemoradiotherapy was provided. Patient currently is free of disease. This is to the best of our knowledge the first report in the world literature of a successfully treated case of “primary mediastinal sarcomas presenting as SVC syndrome.” Patient is under regular surveillance at our clinic and remains free of recurrence one year after treatment completion.

## 1. Introduction

Soft tissue sarcomas (STS) are rare malignant tumors comprising less than 1% of all malignant neoplasm. Among thoracic malignancies, the majority happen to be carcinomas, and the proportion of STS among thoracic malignancies is estimated to be as low as 0.01% [1]. Synovial sarcomas are a rare variety of STS which combine features of both sarcomas and carcinomas. Synovial sarcomas usually are observed to occur in the extremities of young adults. Synovial sarcoma presenting in the mediastinum is exceedingly rare, with only a few reported cases in the world literature [2–7].

The current case report is possibly the first in the world of a patient with SVC obstruction due to a mediastinal synovial sarcoma who responded successfully to treatment with a combined modality approach utilizing neoadjuvant chemotherapy, surgery, postoperative chemotherapy, and radiotherapy. The patient has been rendered free of disease, currently at one-year follow-up after treatment completion.

## 2. Case Report

A 35-year-old nonsmoker male patient presented to the outpatient department with symptoms of rapidly progressive dyspnoea, facial puffiness, and voice-hoarseness developing over a span of 15 days prior to presentation. There was no history of any haemoptysis, abnormal swelling, breathing difficulty, headache, dizziness, tinnitus, epistaxis, or tuberculosis in the past. His overall past medical and surgical history was uneventful.

On examination, the patient was tachypneic with stable vitals. There was a characteristic plethoric face with periorbital and upper body edema with dilated veins over the anterior wall of the chest and neck. There was no pallor, clubbing, lymphadenopathy, ptosis, or any signs of Horner's syndrome. On thoracic auscultation, there were decreased breath sounds in the third and fourth intercostal spaces in the right parasternal area and infraclavicular areas. The rest of the systemic examination was within normal limits.

His blood investigations at presentation revealed hemoglobin level of 11.5 gm/dL, total leukocyte counts of 8500 cells/mm^3^, and platelet counts of 3,20,000/mm^3^. His serum electrolytes, renal function tests, and liver function tests were within normal range. Germ cell tumor markers like lactate-dehydrogenase (LDH), alpha-fetoprotein (AFP), and *β*-human chorionic gonadotropin (*β*-HCG) levels were within normal limits. Bone marrow examination did not show any malignant marrow infiltration. Serology was negative for human immunodeficiency virus (HIV), viral hepatitis B and C.

**Figure 1:**
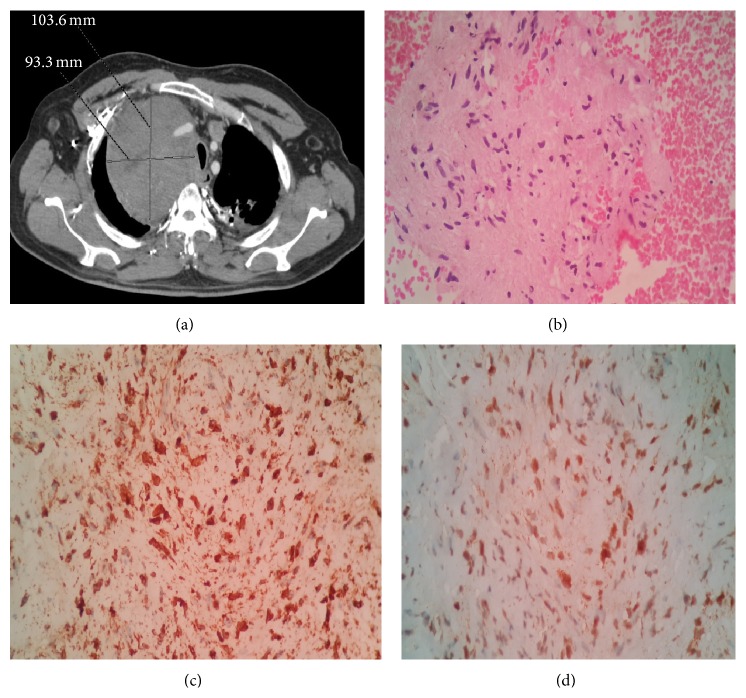
(a) Computed tomography (CT) of the thorax showed a well-defined enhancing soft tissue density lesion of size 10.3 × 9.3 cm involving superior mediastinum, with compression of trachea, SVC, right upper lobe bronchus, and its branches. (b) Histopathological examination of the biopsy specimen from the mediastinal mass was characterized by a monotonous proliferation of tumor cells with oval to spindle, vesicular nuclei surrounded by an indistinct rim of amphophilic to lightly eosinophilic cytoplasm. (c) Immunohistochemical image shows strong vimentin positivity. (d) Immunohistochemical image shows strong S-100 positivity.

Contrast-enhanced computed tomography (CT) of the thorax showed an enhancing well-defined soft tissue density lesion of size 10.3 × 9.3 cm involving superior mediastinum, with compression of trachea, SVC, right upper lobe bronchus, and its branches (Figure 1(a)). The mass was also seen encasing the right brachiocephalic trunk, right subclavian artery, and right common carotid artery.

Histopathological examination of the biopsy specimen was characterized by a monotonous proliferation of tumor cells with oval to spindle, vesicular nuclei surrounded by an indistinct rim of amphophilic to lightly eosinophilic cytoplasm (Figure 1(b)). Immunohistochemistry of the biopsied sample showed positive reactivity to vimentin (Figure 1(c)) and S-100 (Figure 1(d)), epithelial membrane antigen, cytokeratin AE1/AE3, bcl-2, and E-cadherin. Negativity was established for CD34. Cytogenetic analysis by fluorescent in situ hybridization (FISH) sample showed the t(X:18) translocation abnormality. So by combining the histopathological features, IHC, and cytogenetic analysis, diagnosis of monophasic synovial sarcoma was confirmed.

Patient was initially managed symptomatically with diuretics, dexamethasone, oxygen inhalation, and propped up position for symptomatic superior vena cava syndrome. Palliative radiotherapy was omitted owing to the radioresistance of sarcomas. Three cycles of neoadjuvant chemotherapy (ifosfamide 2400 mg/m^2^ on days 1–5 and doxorubicin 37.5 mg/m^2^ on days 1 and 2) were given. Patient tolerated chemotherapy well, and there was partial response to the above mentioned chemotherapeutic drugs according to the RECIST criteria. Surgical excision of the mediastinal mass was carried out via a median sternotomy incision. Postoperative recovery was uneventful.

On cut section, the tumor was composed of gray-white to tan homogeneous soft tissue with focal areas of gelatinous consistency, as well as areas of hemorrhage and necrosis. Histopathological appearance was characterized by a monotonous proliferation of tumor cells with oval to spindle, vesicular nuclei surrounded by an indistinct rim of amphophilic to lightly eosinophilic cytoplasm. Thus the diagnosis of monophasic type of synovial sarcoma of the mediastinum was reconfirmed. The resected surgical margins were free of tumor (R0 resection). Patient was managed postoperatively with adjuvant postoperative radiotherapy (50 Gray in 25 fractions over 5 weeks), followed by three more cycles of chemotherapy. After completion of treatment, the patient is under regular surveillance and for the past one year remains free of recurrence.

## 3. Discussion

The term synovial sarcoma was earlier coined for the tumors arising near the joints. “Synovial sarcoma” owes the name to its resemblance to developing synovial tissue when seen under light microscopy. It however does not arise from the synovial tissue but arises from the pluripotential mesenchymal cells near joint surfaces, tendons, tendon sheaths, juxta-articular membranes, and fascial aponeurosis. These tumors comprise up to 5–10% of all soft tissue sarcomas. The most common age group of occurrence is in the second to fourth decades of life. The most common site of occurrence of these tumors is near the joints and lower extremities. The other sites that may be involved may be the head, neck, trunk, esophagus, intestine, mediastinum, and retroperitoneum. Primary mediastinal synovial sarcoma is a rare malignancy with only a few cases reported so far [2–7].

The differential diagnoses for mediastinal synovial sarcomas would include other rare tumors such as malignant peripheral nerve sheath tumor (MPNST), thymoma, mesothelioma, pulmonary blastoma, and sarcomatoid carcinoma [8].

Since diagnosis based on histopathological appearance in itself is difficult, it is imperative that additional methods such as immunohistochemistry and fluorescent in situ hybridization are necessary. Synovial sarcomas are usually positive for vimentin, epithelial membrane antigen (EMA), bcl-2, CD99 (60–90%), and S-100 (30%). Negativity for myogenin and myoD1 expression is seen, and these markers differentiate synovial sarcomas from rhabdomyosarcomas. Further, the presence of the translocation t(X:18) on fluorescent in situ hybridization is also confirmatory of synovial sarcoma [9–11].

There are three subtypes of synovial sarcoma: the monophasic form (further subdivided into the monophasic spindle cell type, monophasic epithelial cell type, or the monophasic fibrous type), poorly differentiated form, and biphasic form. Monophasic type may be misdiagnosed as leiomyosarcoma, fibrosarcoma, hemangiopericytoma, and malignant peripheral nerve sheath tumor; thus the use of immunohistochemistry will be very much required to confirm a diagnosis. Poorly differentiated variant of synovial sarcoma can be diagnosed on the basis of expression of CD56 and CD59. Biphasic form is histologically composed of two sharply contrasted types of tissues: one of synovial element, the other of fibromatous element. Hence, the biphasic synovial sarcoma can be very easily identified based on characteristic histopathological findings alone [9, 12].

Thus, adequate tissue biopsy will be required for definitive diagnosis based on morphology, special staining, and chromosomal studies. Usually 90% of the synovial sarcomas demonstrate translocation involving fusion of two genes* SYT* located on chromosome 18q11 and* SSX 1*,* SSX2* or* SSX 4* located on Xp11 breakpoint [13].

Synovial sarcomas traditionally have been regarded as being highly aggressive. The most common site of metastasis is lung; however other sites that are less commonly involved include lymph nodes, bone, and bone marrow. Important factors of prognostic significance include the presence of distant metastasis, tumour size (>5 cm), resection margin status, and degree of histological differentiation. Achieving negative surgical margins is highly important, and in case of gross residual disease, reexcision should always be considered. Radiotherapy and adjuvant chemotherapy can enhance the likelihood of disease control.

When complete surgical resection is not possible due to factors such as proximity of tumor to critical structures, maximum possible resection followed by adjuvant radiotherapy is the favored treatment [5]. Synovial sarcoma is one of the few sarcoma subtypes that has a considerable sensitivity to chemotherapy with reported response rates of 30–55%. Two nonrandomized studies had suggested that high dose chemotherapy using agents such as ifosfamide, cisplatin, and doxorubicin may improve disease-free and overall survival rates [14–18].

Patients with unresectable localized disease can be managed with the aim of curing by the use of neoadjuvant chemotherapy and/or radiotherapy so as to obtain a regression which may allow subsequent surgical resection [19]. In our case the primary tumor mass was large enough, making it inoperable. After stabilizing the patients general condition, three cycles of doxorubicin-ifosfamide based chemotherapy were given, and there was partial response to the above mentioned chemotherapeutic drugs according to RECIST criteria. Surgical excision of the mediastinal mass was carried out with median sternotomy incision, and all the margins were free of tumor (R0 resection). Patient was managed postoperatively with radiotherapy (50 Gray in 25 fractions) and three more cycles of doxorubicin-ifosfamide chemotherapy. Patient is under regular surveillance at our clinic for any recurrence for one year without any signs and symptoms of recurrence. This case report makes an evidence of early diagnosis and treatment can make a venue available for resectable as well as for nonresectable tumours so as to achieve complete cure. This case report emphasizes the importance of prompt clinical suspicion, accurate histopathological diagnosis, and use of appropriate immunohistochemical markers and specific chromosomal translocations in the diagnosis of this unusual tumor in an unusual site. This case also illustrates the successful outlook with the use of a combined modality approach for a synovial sarcoma located in a location as difficult as the mediastinum.

## References

[1] Salter D. M. (2006). Pulmonary and thoracic sarcomas. *Current Diagnostic Pathology*.

[2] Jeganathan R., Davis R., Wilson L., McGuigan J., Sidhu P. (2007). Primary mediastinal synovial sarcoma. *Ulster Medical Journal*.

[3] Balieiro M. A., Lopes A. J., Costa B. P. (2013). The surprising outcome of a giant primary mediastinal synovial sarcoma treated with neoadjuvant chemotherapy. *Journal of Thoracic Disease*.

[4] Salah S., Al-Ibraheem A., Daboor A., Al-Hussaini M. (2013). Synovial sarcoma presenting with huge mediastinal mass: a case report and review of literature. *BMC Research Notes*.

[5] Henninger B., Freund M., Zelger B. (2009). Primary mediastinal synovial sarcoma: a case report and review of the literature. *Cases Journal*.

[6] Suster S., Moran C. A. (2005). Primary synovial sarcomas of the mediastinum: a clinicopathologic, immunohistochemical, and ultrastructural study of 15 cases. *The American Journal of Surgical Pathology*.

[7] Pal M., Ghosh B. N., Roy C., Manna A. K. (2010). Posterior mediastinal biphasic synovial sarcoma in a 12 year-old boy: a case report and review of literature. *Journal of Cancer Research and Therapeutics*.

[8] Aubry M.-C., Bridge J. A., Wickert R., Tazelaar H. D. (2001). Primary monophasic synovial sarcoma of the pleura: five cases confirmed by the presence of SYT-SSX fusion transcript. *American Journal of Surgical Pathology*.

[9] Korula A., Shah A., Philip M. A. (2009). Primary mediastinal synovial sarcoma with transdiaphragmatic extension presenting as a pericardial effusion. *Singapore Medical Journal*.

[10] Cessna M. H., Zhou H., Perkins S. L. (2001). Are Myogenin and MyoD1 expression specific for rhabdomyosarcoma? A study of 150 cases, with emphasis on spindle cell mimics. *The American Journal of Surgical Pathology*.

[11] Pelmus M., Guillou L., Hostein I., Sierankowski G., Lussan C., Coindre J.-M. (2002). Monophasic fibrous and poorly differentiated synovial sarcoma: immunohistochemical reassessment of 60 t(X;18)(SYT-SSX)-positive cases. *The American Journal of Surgical Pathology*.

[12] Suster S., Moran C. A. (2005). Primary synovial sarcomas of the mediastinum: a clinicopathologic, immunohistochemical, and ultrastructural study of 15 cases. *The American Journal of Surgical Pathology*.

[13] Kwon O. Y., Lee S. K., Cho M. K., Kim Y. J. (2007). A case of biphasic synovial sarcoma of frontal bone in an elderly patient. *Journal of Korean Neurosurgical Society*.

[14] van Glabbeke M., van Oosterom A. T., Oosterhuis J. W. (1999). Prognostic factors for the outcome of chemotherapy in advanced soft tissue sarcoma: an analysis of 2,185 patients treated with anthracycline-containing first-line regimens—a European organization for research and treatment of cancer soft tissue and bone sarcoma group study. *Journal of Clinical Oncology*.

[15] Singer S., Baldini E. H., Demetri G. D., Fletcher J. A., Corson J. M. (1996). Synovial sarcoma: prognostic significance of tumor size, margin of resection, and mitotic activity for survival. *Journal of Clinical Oncology*.

[16] Kampe C. E., Rosen G., Eilber F. (1993). Synovial sarcoma: a study of intensive chemotherapy in 14 patients with localized disease. *Cancer*.

[17] Rosen G., Forscher C., Lowenbraun S. (1994). Synovial sarcoma. Uniform response of metastases to high dose ifosfamide. *Cancer*.

[18] Spillane A. J., A'Hern R., Judson I. R., Fisher C., Thomas M. J. M. (2000). Synovial sarcoma: a clinicopathologic, staging, and prognostic assessment. *Journal of Clinical Oncology*.

[19] Balieiro M. A., Lopes A. J., Costa B. P. (2013). The surprising outcome of a giant primary mediastinal synovial sarcoma treated with neoadjuvant chemotherapy. *Journal of Thoracic Disease*.

